# Supplementary material for: Influence of Surface Cleaning on Quantum Efficiency, Lifetime and Surface Morphology of p-GaN:Cs Photocathodes
Source: Micromachines (Basel). 2022 May 29;13(6):849. doi: 10.3390/mi13060849 (PMC9227593; doi:10.3390/mi13060849)
Supplement: Supplementary file 1 [file micromachines-13-00849-s001.zip › micromachines-1740287-supplementary.pdf]

Supplementary Information

p-GaN on sapphire

Surface morphology after thermal treatment and activation

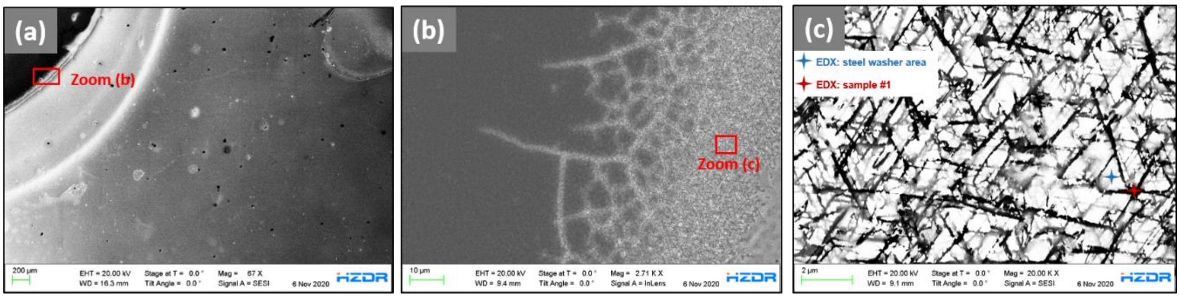

Figure S1. SEM images of p-GaN:Cs on sapphire photocathode (sample A1), overview (S1**a**), Zoom in the area of the steel washer (S1**b**) and a Zoom with higher magnification (S1**c**), where EDX measurements (S2) were taken.

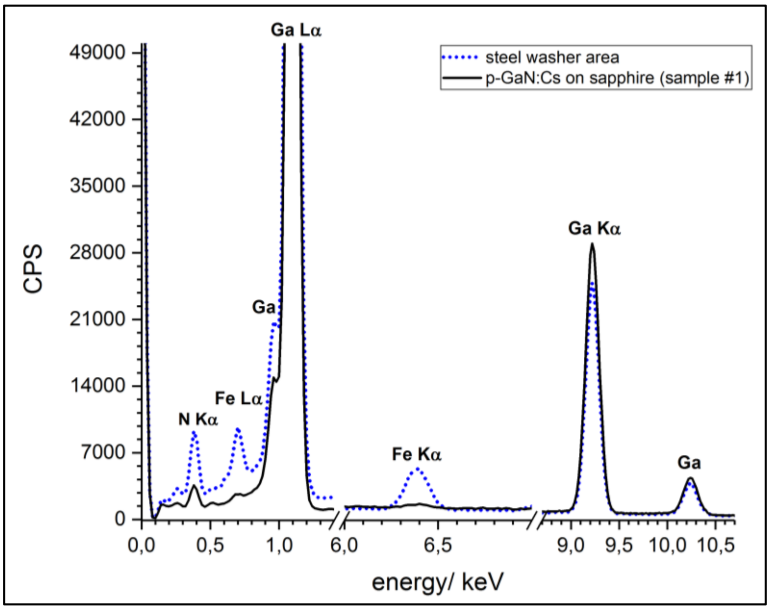

Figure S2. EDX measurement of p-GaN:Cs on sapphire photocathode (sample A1) from SEM image S1c, showing the difference between the bright (steel washer area) and the dark area (original p-GaN:Cs photocathode surface).

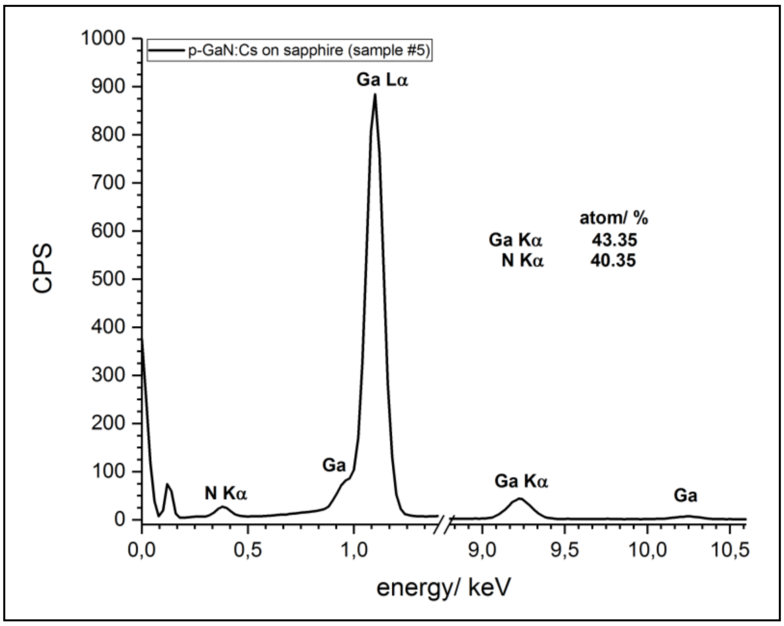

Figure S3. EDX measurement of p-GaN:Cs on sapphire photocathode (sample A5), showing the homogeneity of the photocathode surface with a Ga:N ratio of 1:1.

Cesium was not detectable here because EDX analysis gives good quantitative results on bulk measurements from several µm depth. Therefore, EDX is not the choice for thin film analysis in the range of a few atomic layers.

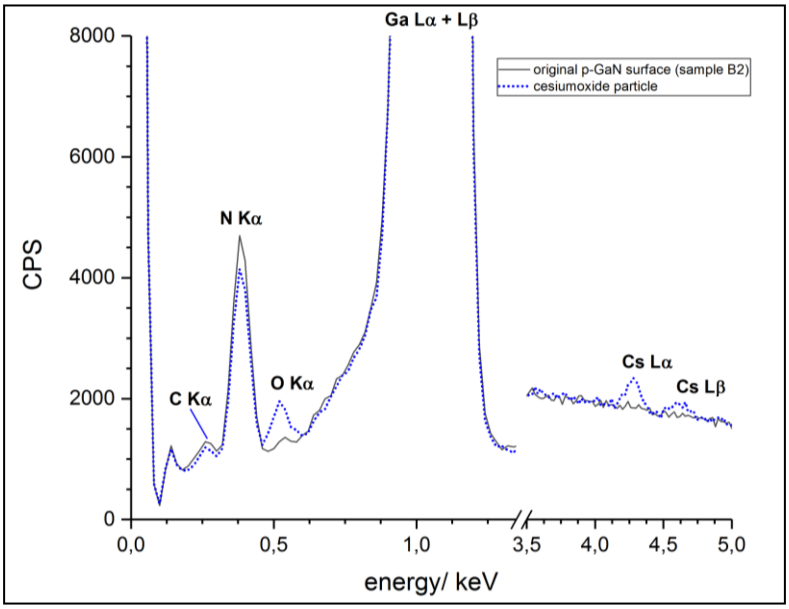

Figure S4. EDX spectra of p-GaN:Cs on silicon photocathode (sample B2), showing that the round particle contains cesium and oxygen (blue dots), compared to the p-GaN:Cs photocathode surface (black line).

Figure S4 show that characteristic signals from cesium and a higher amount of oxygen were found when EDX is focused on the round particles on the sample B2 surface. Therefore, we can identify the round particles as cesium oxide or cesium hydroxide.
